# Inflammation and corticosteroid responsiveness in ex-, current- and never-smoking asthmatics

**DOI:** 10.1186/1471-2466-13-58

**Published:** 2013-09-22

**Authors:** Eef D Telenga, Huib A M Kerstjens, Nick H T ten Hacken, Dirkje S Postma, Maarten van den Berge

**Affiliations:** 1 Department of Pulmonary Diseases, University Medical Center Groningen, Groningen, Netherlands; 2 Groningen Research Institute for Asthma and COPD, University of Groningen, University Medical Center Groningen, Groningen, Netherlands

**Keywords:** Asthma, Smoking, Corticosteroid responsiveness, Lung function

## Abstract

**Background:**

It has been suggested that smoking asthmatics benefit less from corticosteroid treatment than never-smoking asthmatics. We investigated differences in blood and sputum inflammatory profiles between ex-, current-, and never-smokers and assessed their ICS treatment response after 2-week and 1-year treatment.

**Methods:**

We analyzed FEV_1_, PC_20_ methacholine and PC_20_ AMP, (differential) cell counts in sputum and blood in ex-, current- and never-smokers at baseline (n=114), after 2-week treatment with fluticasone 500 or 2000 μg/day (n=76) and after 1-year treatment with fluticasone 500 μg/day or a variable dose of fluticasone based on a self-management plan (n=64).

**Results:**

A total of 114 patients were included (29 ex-, 30 current- and 55 never-smokers. At baseline, ex- and current-smokers had less eosinophils in sputum and blood than never-smokers. Blood neutrophil counts were higher in current- than in never-smokers. A higher number of cigarettes smoked daily was associated with lower blood and sputum eosinophils. After 2-week ICS treatment, FEV_1_ %predicted improved less in current-smokers than never-smokers (2.4% versus 8.1%, p=0.010) and ex-smokers tended to improve less than never-smokers (4.1%, p=0.067). In contrast, no differences in ICS treatment response in lung function or inflammatory cells were found between the three groups after 1 year.

**Conclusions:**

Ex- and current-smokers have less eosinophils and more neutrophils in their sputum and blood than never-smokers. Although ex- and current-smokers have a reduced short-term corticosteroid treatment response, we did not find a difference in their long-term treatment response.

## Background

Asthma is a chronic inflammatory airway disease in which a variety of inflammatory cells and mediators play a role. Inhaled corticosteroids (ICS) are the cornerstone of treatment, since they exert broad anti-inflammatory effects. They have been shown to improve symptoms and lung function as well as bronchial hyperresponsiveness and markers of airway inflammation in blood, induced sputum and bronchial biopsies [1]. In addition, the use of ICS reduces the number of asthma exacerbations [2].

About 20-30% of asthma patients smoke and another 20-40% are ex-smokers [3-6]. Current-smokers appear to have a different airway inflammatory profile than never-smokers, with less eosinophilic and more neutrophilic inflammation [7-12]. Thus far, very little is known about the inflammatory profile of ex-smokers.

The few studies investigating the effects of smoking on the short-term efficacy of oral or inhaled corticosteroid treatment in asthma, demonstrate that the forced expiratory volume in one second (FEV_1_) improves significantly in never-smokers, but not in current-smokers [7,13-15]. However, none of these studies found statistically significant differences in improvement in FEV_1_ when directly comparing never- and current-smokers. The only study that included ex-smokers, showed no improvement in FEV_1_ or asthma control after 2-week oral corticosteroid treatment in ex- and current-smokers [15].

We aimed to investigate whether ex-, current- and never-smokers with asthma have different inflammatory profiles and if current number of cigarettes or packyears smoked affect this. Furthermore, we assessed whether the short- and long-term responsiveness to corticosteroids after 2-week and 1-year treatment is different between ex-, current- and never-smoking asthmatics. We have analyzed this in a relatively large group of 114 well-characterized patients with allergic, mild to moderately severe asthma [16].

## Methods

### Patients

Patients with a diagnosis of asthma, 18–65 years old, were included if they met the following criteria: provocative concentration of methacholine inducing a 20% fall of FEV_1_ (PC_20_ methacholine) ≤8 mg/ml, at least one positive skin-prick test out of 17 common aero-allergens, reversibility to salbutamol 200 μg ≥9% of the predicted FEV_1_ and the ability to expectorate sputum after hypertonic saline inhalation. This study was conducted in accordance with the amended declaration of Helsinki and the study was approved by the medical ethics committee of the University Medical Center Groningen and all participants gave their written informed consent.

### Study design

**Figure 1:**
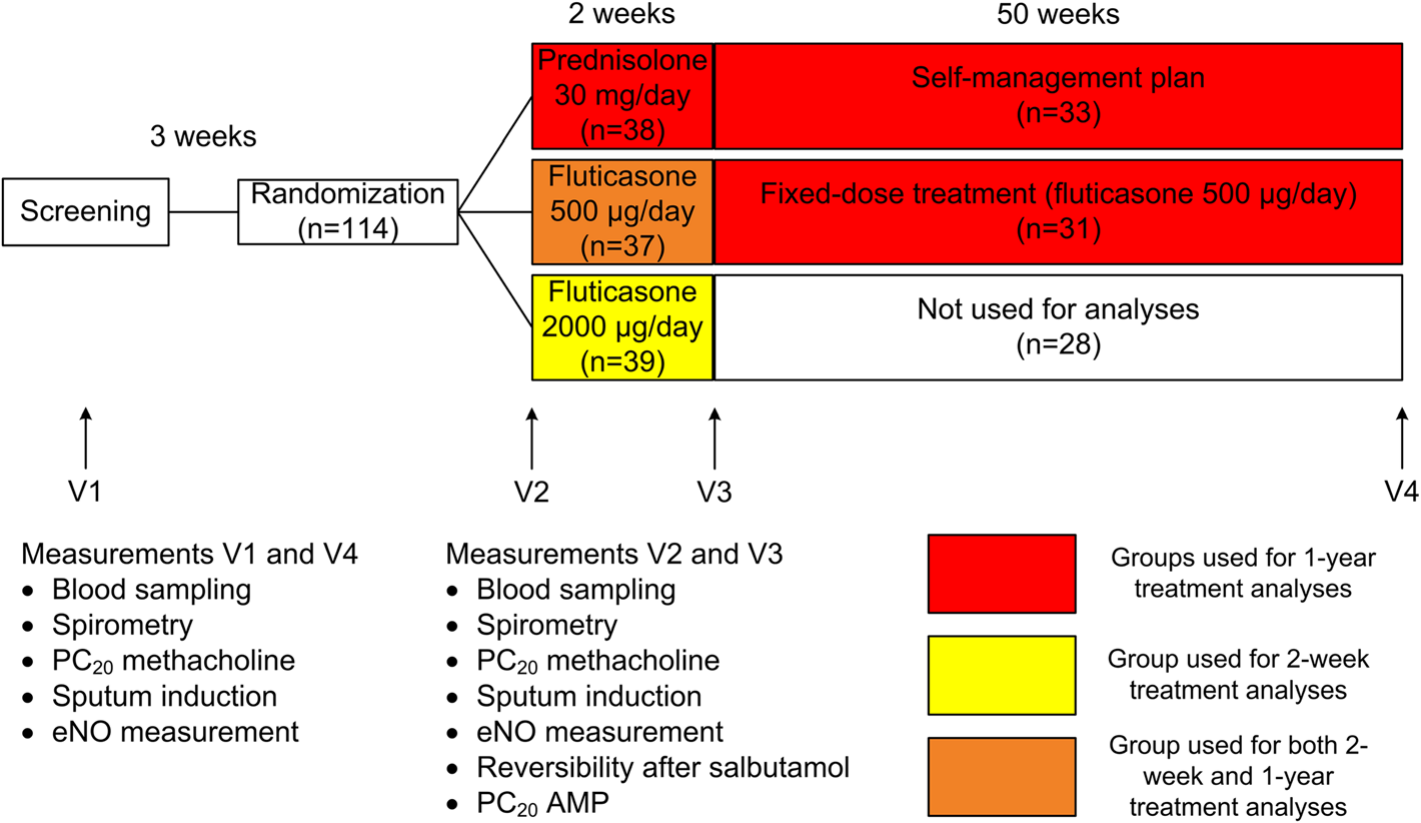
**Flow**-**chart of the study.** FEV_1_ = forced expiratory volume in one second, PC_20_ = provocative concentration causing a 20% fall in FEV_1_, AMP = adenosine-5′-monophosphate.

Figure 1 shows the outline of the study. ICS were tapered before enrollment in the study, as described in the original manuscript [16]. After discontinuation of ICS completely for three weeks, or earlier, if they experienced symptoms of an asthma exacerbation, patients were randomized to 3 treatment arms, with minimization according to smoking status, age, previous dose of ICS, FEV_1_ %predicted, reversibility after 200 μg of salbutamol, PC_20_ methacholine, and serum IgE. Patients were first treated for 2 weeks with either prednisolone 30 mg/day, fluticasone 500 μg/day or fluticasone 2000 μg/day via Diskhaler, followed by another 50 weeks of treatment as follows.

The prednisolone 30 mg/day group was treated according to a self-management plan. They first received fluticasone 200 μg/day and were instructed to change the dose according to a self-management plan (see Additional file 1: Table S1). The fluticasone 500 μg/day group continued with the same dose for another 50 weeks. The fluticasone 2000 μg/day arm followed a program with step-down and eventually complete discontinuation of corticosteroids. The latter is not in agreement with the current guidelines and therefore this arm was removed from our long-term analyses. During the first 2 weeks, the study had a double-blind, double-dummy design, followed by 50 weeks open label treatment. Rescue medication consisted of salbutamol 400 μg via Diskhaler. No other concomitant pulmonary medication was allowed.

Patients with an exacerbation were treated with a standardized 7-day course of oral prednisolone. Patients were withdrawn if they required >1 hospitalization, >4 courses of oral prednisolone or >2 courses within 3 months. Requirement of >2000 μg fluticasone in the self-management group additionally led to withdrawal.

### Lung function and bronchial hyperresponsiveness

FEV_1_ was measured with a calibrated, water-sealed spirometer according to standardized guidelines before and 20 minutes after 200 μg of salbutamol [17]. Provocation tests were performed using a 2-minute tidal breathing method, adapted from Cockcroft and coworkers [18]. After an initial nebulized saline challenge, subjects inhaled doubling concentrations of the provocative agent (methacholine-bromide 0.038 to 19.6 mg/l or adenosine-5′-monophosphate (AMP) 0.04 to 320 mg/ml) at 5 minute intervals. All calculations of PC_20_ were performed with a base-2 logarithm, reflecting doubling concentrations and normalizing the distribution.

### Sputum induction and processing

Sputum was induced by inhalation of hypertonic saline as previously described [16]. Fifteen minutes after salbutamol (200 μg) inhalation, hypertonic saline (3%, 4%, and 5%) was nebulized for each concentration during 7 minutes. Whole samples were processed according to the method of Fahy *et al*. with some modifications [19].

Cell counts in blood were performed by flow cytometry. Eosinophilic cationic protein (ECP) in serum and sputum were measured with a fluoroenzyme assay (ImmunoCAP ECP, Pharmacia, Uppsala, Sweden). Exhaled nitric oxide (NO) was measured by tidal breathing method using a chemiluminescence analyzer (CLD 700 AL, ECO physics, Switzerland) as described previously [16].

### Statistical methods

In case of non-normal distribution, log-transformation was performed to obtain normally distributed variables. Baseline differences between ex-, current- and never-smokers were tested by analysis of variance (ANOVA), Kruskal-Wallis or Chi-square test. If a significant difference between the three groups was found, we performed post-hoc tests with Holm’s Bonferroni correction for multiple testing. Short-term treatment effects were analyzed only in the two groups using ICS (i.e. fluticasone 2000 μg/day or 500 μg/day). To test for changes after treatment within a group (i.e. ex-, current- or never-smokers), we performed paired t-tests. To test for differences in corticosteroid treatment responsiveness between groups, we performed linear regression analyses with change from baseline of each variable as outcome variable and smoking status as the predictor variable and age, gender and type of treatment as covariates. In addition, we adjusted for the baseline value of each variable, since this has been shown to be one of the major predictors of treatment response [20]. To test the effect of current and cumulative smoke exposure on baseline differences and treatment response, we performed linear regression analyses with either the number of cigarettes/day or packyears as predictor variables. We added age as a covariate in these analyses. The reported correlation coefficient (b) signifies the change in an outcome variable (e.g. FEV_1_) for every unit increase of the predictor variable (e.g. cigarettes/day). In all regression analyses with absolute FEV_1_ we corrected for age, gender and height.

## Results

### Patient characteristics

114 patients were included, 29 ex-smokers, 30 current-smokers and 55 never-smokers. Their baseline characteristics, after tapering of ICS (visit 2), are presented in Table 1. During the ICS tapering period, 16 patients returned to the hospital earlier due to symptoms compatible with an asthma exacerbation. From these 16 patients, 6 still used ICS at the start of the treatment period (2 ex-smokers, 2 current-smokers and 2 never-smokers) with a median beclomethasone equivalent dose of 450 μg/day (range 400 – 800 μg/day); the remaining 10 patients had discontinued ICS completely for a median period of 12 days (range 2 – 21 days). Ex-smokers had a median smoking cessation period of 7 years (interquartile range (IQR) 1.5 -15.5 years) and had smoked a median of 6.9 packyears (IQR 3.5 – 20.8). Current-smokers had smoked 7.4 packyears (IQR 2.5 – 14.1) and smoked a median of 8.0 cigarettes/day (IQR 4.6 – 15.0).

**Table 1:** **Differences in clinical and inflammatory variables between ex**-, **current**- **and never**-**smokers at baseline**

	**Ex**-**smokers (n=29)**	**Current-smokers (n=30)**	**Never-smokers (n=55)**	**p-value**
Age (years)	38 (28, 41)	27^‡^ (25, 37)	25^‡^ (25, 35)	0.001
Gender (male/female)^#^	13 / 16	10 / 20	16 / 39	0.349
Daily number of cigarettes	–	8.0 (4.6, 15.0)	–	
Packyears (number)	6.9 (3.5, 20.8)	7.4 (2.5, 14.1)	–	
Duration of smoking cessation (years)	7.0 (1.5, 15.5)	–	–	
Still using ICS after tapering (yes/no)^#^	2 / 27	2 / 28	2 / 53	0.645
Treatment^#^ (prednisolone/FP500/FP2000)	10 / 11 / 8	11 / 6 / 13	17 / 20 / 18	0.508
FEV_1_ (L)	2.9 (2.3, 3.4)	2.8 (2.4, 3.4)	3.0 (2.3, 3.4)	0.911
FEV_1_ (%predicted)	79 (68, 89)	78 (70, 91)	82 (62, 94)	0.957
Reversibility (%predicted)	11 (9, 17)	11 (9, 15)	13 (9, 18)	0.150
PC_20_ methacholine (mg/ml)^§^	0.7 (0.06, 7.9)	0.8 (0.03, 7.3)	0.4 (0.02, 7.8)	0.123
PC_20_ AMP (mg/ml)^§^	10.3 (0.2, 640)	7.2 (0.2, 640)	3.6 (0.02, 640)	0.179
Sputum eosinophils (%)	2.8^*^ (1.1, 6.0)	4.7^*^ (0.8, 10.7)	7.7 (3.8, 14.3)	0.015
Blood eosinophils (10^9^/L)	0.27^*^ (0.14, 0.43)	0.28^*^ (0.15, 0.41)	0.44 (0.34, 0.61)	0.001
Sputum ECP (μg/L)	33 (19, 124)	67 (16, 126)	49 (17, 163)	0.979
Serum ECP (μg/L)	10^*^ (8, 17)	14 (9, 23)	22 (12, 29)	0.001
Sputum neutrophils (%)	39 (22, 53)	42 (26, 65)	29 (20, 50)	0.175
Blood neutrophils (10^9^/L)	3.9 (3.0, 4.6)	4.1^*^ (3.4, 5.3)	3.1 (2.6, 4.1)	0.003
Exhaled NO (ppb)	15 (11, 21)	12 (6, 17)	16 (12, 21)	0.058

Values are presented as medians with interquartile ranges, unless stated otherwise, # = number, § geometric mean (range), prednisolone = prednisolone 30 mg once daily, *FP*500 fluticasone propionate 500 μg/day, *FP*2000 fluticasone propionate 2000 μg/day, *FEV*_1_ forced expiratory volume in one second, *PC*_20_ provocative concentration causing a 20% fall in *FEV*_1_, *AMP* adenosine-5′-monophosphate, *ECP* eosinophilic cationic protein, *NO* nitric oxide, ppb parts per billion, * = p<0.05 compared to never-smokers with Holm’s Bonferroni correction, ‡ = p<0.05 compared to ex-smokers with Holm’s Bonferroni correction.

### Baseline differences between ex-, current- and never-smokers

Ex-smoking asthmatics were significantly older than current- and never-smokers (median 38 versus 27 and 25 years, respectively; p=0.001). Sputum eosinophil percentages and blood eosinophil counts were significantly lower in ex- and current-smokers than in never-smokers. Serum ECP, a marker of eosinophil activation, was significantly lower in ex- than never-smokers. Blood neutrophil counts were higher in current- than in never-smokers. Blood neutrophil counts of ex-smokers were between those of never- and current-smokers, but not significantly different from either group. FEV_1_, reversibility to salbutamol, bronchial hyperresponsiveness to methacholine or AMP and exhaled NO were comparable between ex-, current- and never-smokers.

### Association between current and cumulative smoke exposure and baseline clinical and inflammatory parameters

In current-smokers, a higher number of cigarettes smoked daily was associated with lower sputum eosinophil percentages, blood eosinophil counts and serum ECP (Table 2). Furthermore, it was associated with less severe bronchial hyperresponsiveness to both methacholine and AMP (0.1 doubling dose per cigarette/day for methacholine and 0.2 doubling concentrations per cigarette/day for AMP). In ex- and current-smokers, a higher number of packyears was associated with a lower FEV_1_ %predicted (p=0.034).

**Table 2:** Association between the amount of smoke exposure, as reflected by the number of cigarettes smoked daily and number of packyears and clinical and inflammatory variables at baseline

	**Cigarettes/day**		**Packyears**	
	**b**	**p-value**	**b**	**p-value**
FEV_1_ (L)	0.13	0.508	–0.01	0.450
FEV_1_ (%predicted)	0.06	0.897	–0.31	0.034
Reversibility (%predicted)	–0.22	0.162	–0.00	0.968
PC_20_ methacholine (doubling concentrations)	0.11	0.050	0.04	0.123
PC_20_ AMP (doubling concentrations)	0.19	0.031	0.02	0.593
Sputum eosinophils (%)*	–0.06	0.024	–0.01	0.496
Blood eosinophils (10^9^/L)*	–0.02	0.021	0.00	0.645
Sputum ECP (μg/L)*	–0.04	0.313	–0.00	0.839
Serum ECP (μg/L)*	–0.04	0.025	0.00	0.829
Sputum neutrophils (%)*	–0.00	0.779	0.00	0.713
Blood neutrophils (10^9^/L)*	0.00	0.673	–0.00	0.338
Exhaled NO (ppb)	–0.09	0.708	0.10	0.254

b = unstandardized regression coefficient, *FEV*_1_ forced expiratory volume in one second, *PC*_20_ provocative concentration causing a 20% fall in *FEV*_1_, *AMP* adenosine-5′-monophosphate, *ECP* eosinophilic cationic protein, *NO* nitric oxide, *ppb* parts per billion, * variable log-transformed.

### Short-term efficacy of ICS treatment in ex-, current- and never-smokers

**Figure 2:**
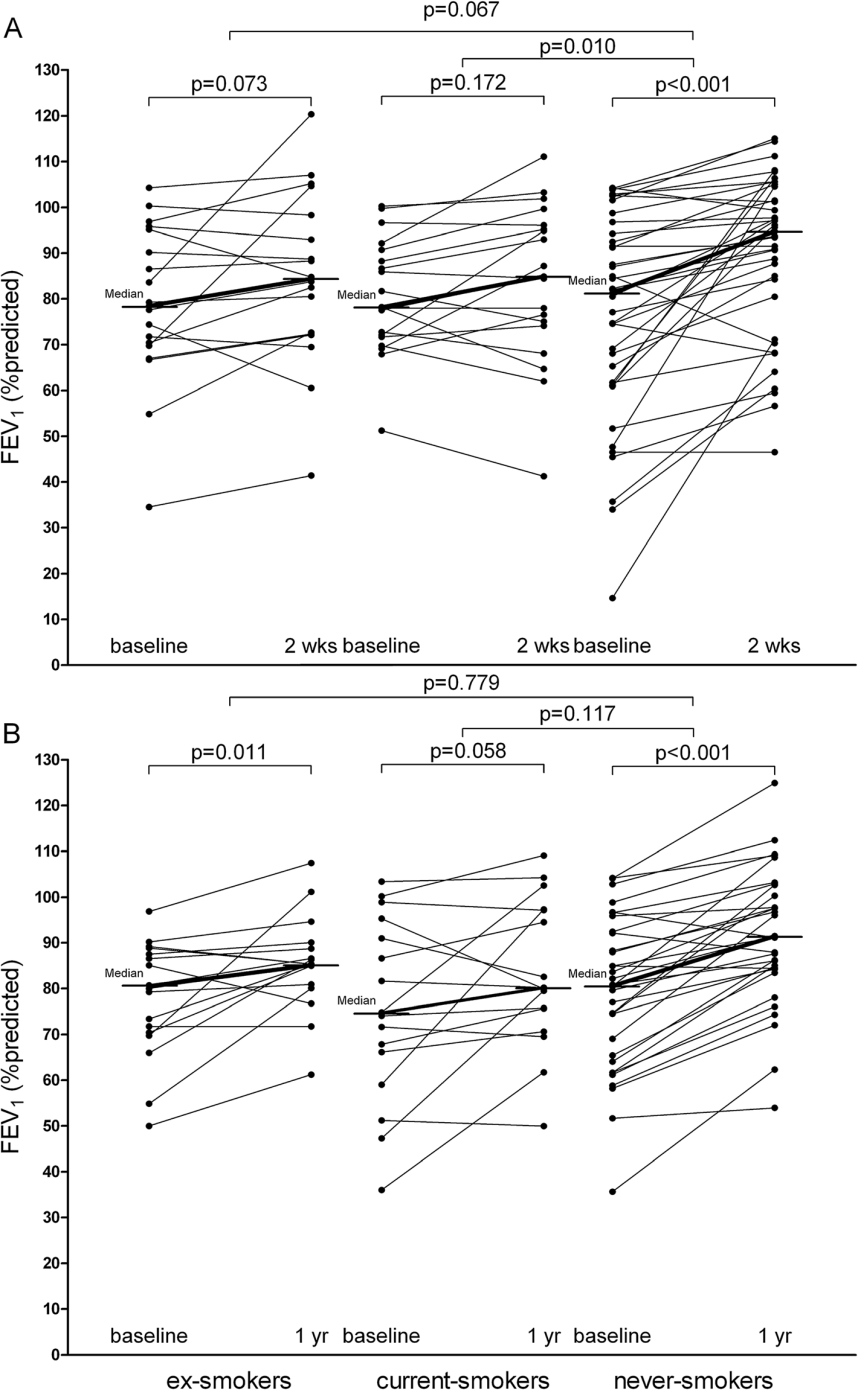
**Change in FEV**_**1**_** % predicted after 2-week and 1-year treatment with ICS. A** = 2-week treatment, **B** = 1-year treatment, FEV_1_ = forced expiratory volume in one second.

76 patients were treated with fluticasone 2000 μg/day or 500 μg/day. After 2-week treatment, FEV_1_ %predicted levels improved significantly in never-smokers (8.1%, p<0.001, Table 3), but not in ex- or current-smokers (4.1%, p=0.073 and 2.4%, p=0.172 respectively). The magnitude of improvement in FEV_1_ %predicted was significantly lower in current- than in never-smokers (p=0.010, Figure 2A) and tended to be lower in ex- than in never-smokers (p=0.067). Sputum eosinophil percentages and ECP concentrations improved less in current- than never-smokers and tended to improve less in ex- than never-smokers. No significant differences in short-term ICS-induced improvements in bronchial hyperresponsiveness and exhaled NO were observed between the three groups. A higher number of packyears smoked was associated with less improvement in FEV_1_ %predicted (-0.55% per packyear, p=0.025, Additional file 1: Table S1). The number of cigarettes smoked daily was not associated with the short-term ICS response in current-smokers.

**Table 3:** Treatment differences between ex-, current- and never- smokers after 2-week ICS treatment

	**Ex-smokers (n=19)**	**p-value**	**Current-smokers (n=19)**	**p-value**	**Never-smokers (n=38)**	**p-value**
Age (years)	38 (28, 44)		27 (25, 42)		25 (25, 36)	
Gender (male/female)	9 / 10		4 / 15		12 / 26	
Treatment (FP500/FP2000)	11 / 8		6 / 13		20 / 18	
ΔFEV_1_ (L)	0.14 (-0.07, 0.31)	0.051	0.08^*^ (-0.05, 0.32)	0.113	0.30 (0.18, 0.77)	<0.001
ΔFEV_1_ (%predicted)	4.1^#^ (-2.1, 9.2)	0.073	2.4^*^ (-4.7, 8.7)	0.172	8.1 (4.6, 20.4)	<0.001
ΔReversibility (%predicted)	-4.8 (-8.1, -0.3)	0.022	-4.3 (-7.9, 2.3)	0.025	-6.8 (-9.7, -2.9)	<0.001
ΔPC_20_ methacholine (doubling concentrations)	1.3^#^ (0.4, 2.0)	0.001	1.4 (0.3, 2.4)	0.001	2.3 (1.1, 3.2)	<0.001
ΔPC_20_ AMP (doubling concentrations)	3.1 (0.4, 5.7)	0.001	0.9^#^ (0.1, 5.6)	0.007	5.1 (2.2, 6.4)	<0.001
ΔSputum eosinophils (%)	-1.4 (-5.7, -0.7)	<0.001	-1.0^‡^ (-4.5, 0.0)	0.004	-6.3 (-14.5, -2.2)	<0.001
ΔBlood eosinophils (10^9^/L)	-0.05 (-0.16, 0.02)	0.018	-0.02 (-0.15, 0.03)	0.060	-0.16 (-0.28, -0.01)	0.002
ΔSputum ECP (μg/L)	-12 (-82, -1)	0.022	-10^*§^ (-48, 9)	0.219	-31 (-135, -1)	<0.001
ΔSerum ECP (μg/L)	-1.3 (-4.5, 2.1)	0.564	-2.4^#^ (-15.0, -4.2)	0.303	-6.9 (-14.6, -1.0)	<0.001
ΔSputum neutrophils (10^9^/L)	-3.3 (-17.4, 7.8)	0.270	-2.7^§^ (-15.0, 4.2)	0.496	0.5 (-7.8, 6.3)	0.382
ΔBlood neutrophils (10^9^/L)	0.31 (-0.57, 0.78)	0.625	0.27^§^ (-0.47, 0.97)	0.902	0.22 (-0.26, 0.79)	0.563
ΔExhaled NO (ppb)	-3.3 (-6.8, 0.00)	0.039	-3.6 (-6.8, 3.1)	0.248	-5.1 (-8.4, -2.1)	<0.001

Values are presented as median change from baseline with interquartile range. *ICS* inhaled corticosteroids, *FP*500 fluticasone propionate 500 μg/day, *FP*2000 fluticasone propionate 2000 μg/day, *FEV*_1_ forced expiratory volume in one second, *PC*_20_ provocative concentration causing a 20% fall in *FEV*_1_, *AMP* adenosine-5′-monophosphate, *ECP* eosinophilic cationic protein, *NO* nitric oxide, *ppb* parts per billion,* Significantly different from never-smokers (p<0.05), # Trend for difference from never-smokers (0.05<p≤0.1), ‡ Significantly different from ex-smokers (p<0.05), § Trend for difference from ex-smokers (0.05<p≤0.1).

### Long-term efficacy of ICS treatment in ex-, current- and never-smokers

Data from 64 patients treated for 1-year with fluticasone 500 μg/day or a variable dose of fluticasone according to the self-management plan were available (Table 4). In the self-management group, the median daily dose of fluticasone over the 50 week period was 275 μg/day (range 200–1375 μg/day), which was significantly lower than the 500 μg/day used by the fixed-dose group. The level of FEV_1_ %predicted improved significantly in ex- and never-smokers, (5.1%, p=0.011 and 10.2%, p<0.001 respectively) and tended to improve in current-smokers (3.1%, p=0.058). There was no significant difference in the magnitude of improvement in FEV_1_ between the three groups (Figure 2B). The treatment-induced changes in PC_20_ methacholine and numbers and percentages of inflammatory cells in blood and sputum did also not differ significantly between ex-, current- and never-smokers. A higher number of packyears was associated with less improvement in FEV_1_ %predicted (p=0.032, Additional file 1: Table S2). In addition, the severity of PC_20_ methacholine improved less with a higher number of packyears smoked (p=0.043). The number of cigarettes smoked daily was not associated with the magnitude of improvement in FEV_1_ or PC_20_ methacholine.

**Table 4:** Treatment differences between ex-, current- and never-smokers after 1-year ICS treatment

	**Ex-smokers (n=16)**	**p-value**	**Current-smokers (n=16)**	**p-value**	**Never-smokers (n=32)**	**p-value**
Age (years)	37 (27, 40)		29 (25, 36)		25 (25, 34)	
Gender (male/female)	9 / 12		7 / 10		10 / 27	
Treatment (FP500/self management)	11 / 10		6 / 11		20 / 17	
ΔFEV_1_ (L)	0.15 (0.00, 0.60)	0.010	0.17 (-0.07, 0.82)	0.052	0.35 (0.22, 0.71)	<0.001
ΔFEV_1_ (%predicted)	5.1 (0.4, 13.9)	0.011	3.1 (-1.7, 21.5)	0.058	10.2 (6.4, 20.1)	<0.001
ΔPC_20_ methacholine (doubling concentrations)	2.7 (1.5, 5.7)	0.002	2.3 (1.4, 3.1)	<0.001	4.4 (2.1, 5.5)	<0.001
ΔSputum eosinophils (%)	-2.7 (-4.5, -0.3)	0.005	-2.0 (-14.3, -0.1)	0.029	-7.0 (11.9, -1.3)	<0.001
ΔBlood eosinophils (×10^9^/L)	-0.04 (-0.05, 0.02)	<0.001	-0.04 (-0.08, 0.05)	<0.001	-0.16 (-0.26, -0.04)	<0.001
ΔSputum ECP (μg/L)	11 (-5, 33)	0.194	-11 (-165, 6)	0.096	-19 (-73, 3)	0.023
ΔSerum ECP (μg/L)	0.1 (-3.1, 2.3)	0.576	-1.8 (-12.8, 2.9)	0.268	-9.2 (-19.3, -4.1)	<0.001
ΔSputum neutrophils (10^9^/L)	8.5 (-18.2, 23.3)	0.126	4.8 (-22.2, 24.3)	0.641	6.9 (-5.1, 24.6)	0.077
ΔBlood neutrophils (10^9^/L)	0.32 (-0.51, 0.70)	<0.001	-0.15 (-0.87, 0.21)	<0.001	-0.40 (-0.78, 0.39)	<0.001
ΔExhaled NO (ppb)	-4.9 (-7.1, 2.1)	0.182	-6.1 (-9.3, 2.9)	0.033	-4.4 (-7.9, -1.4)	0.002

Values are presented as median change from baseline with interquartile range. *ICS* inhaled corticosteroids, *FP*500 fluticasone propionate 500 μg/day, *FEV*_1_ forced expiratory volume in one second, *PC*_20_ provocative concentration causing a 20% fall in *FEV*_1_, *AMP* adenosine-5′-monophosphate, *ECP* eosinophilic cationic protein, *NO* nitric oxide, *ppb* parts per billion.

### Effect of inflammation on improvement in lung function

To investigate if the baseline type and level of inflammation was associated with the corticosteroid treatment response, we analyzed the independent associations between the improvement in FEV_1_ %predicted after 2-week and 1-year ICS treatment and eosinophils in sputum and blood and smoking status. Sputum: higher percentages of sputum eosinophils were significantly associated with a greater improvement in FEV_1_ %predicted both after 2-week and 1-year treatment (b = 0.252, p= 0.005 and b=0.232, p=0.002 respectively, Additional file 1: Table S3), whereas sputum neutrophils were not independently associated with improvement in FEV_1_ %predicted. Blood: higher levels of blood eosinophil and lower levels of blood neutrophils were independently associated with a higher improvement in FEV_1_ %predicted after 2-week ICS treatment (b=0.529, p=0.022 and b=-0.343, p=0.049 respectively, Additional file 1: Table S4). After 1 year ICS treatment blood eosinophil levels were still significantly associated with improvement in FEV_1_ %predicted. Smoking status was not significantly associated with improvement in FEV_1_ %predicted, when inflammation was taken into account (Additional file 1: Tables S3 and S4).

## Discussion

Our study shows that current-smokers with asthma have a different type of inflammation, i.e. they have less eosinophils and more neutrophils in their sputum and blood than never-smokers, even though the severity of airflow obstruction and bronchial hyperresponsiveness is comparable. Moreover, a higher number of cigarettes smoked daily was associated with a lower percentage of eosinophils in sputum, suggesting that the type of airway inflammation may be influenced by the amount of smoke exposure. Interestingly, the inflammatory profile of a group of asthmatics with a median smoking cessation of 7 years was more similar to that of the current-smoking than that of the never-smokers, suggesting that effects of smoking may persist for a long time after smoking cessation in asthmatics. Additionally, we show that current-smokers have a blunted short-term corticosteroid treatment response. Again, ex-smokers are more similar to current-smokers than to never-smokers, with a trend for a blunted response. However, we found no evidence for a blunted response in both ex- and current-smokers on the long-term.

After short-term treatment with ICS, current-smokers had less improvement in FEV_1_ than never-smokers, as reported earlier [7,13,15]. We extend these findings by showing that ex-smokers also tend to respond less to corticosteroid treatment than never-smokers on the short-term. Thus far, the efficacy of corticosteroid treatment in ex-smokers has only been investigated in one study with 15 asthmatic ex-smokers [15]. Comparable to our findings, they observed that the short-term improvement in FEV_1_ after 2-week treatment with oral corticosteroids in ex-smokers was intermediate between current- and never-smokers.

Interestingly, we found that the long-term effects of 1-year ICS treatment were not significantly different in ex- and current-smokers compared to never-smokers. This observation is in line with a study in 492 current- and 2,432 never-smokers, showing that 400 μg/day budesonide or placebo for 3 years was equally effective in current- and never-smokers [21]. Furthermore, in a large, real-life study in 619 asthmatics, the level of improvement in FEV_1_ and asthma control was similar in ex-, current- and never-smokers after 1-year treatment with small particle budesonide/formoterol formulation [22]. Taken together, these findings suggest that ex- and current-smokers with asthma have a lower corticosteroid treatment response on the short-term than never-smoker, whereas the long-term response is similar between the three groups. We extend these observations by showing that 1-year ICS treatment response is not driven by smoking per se. Rather the underlying inflammatory process present drives the ICS response over 1 year, i.e. a better response with higher sputum and blood eosinophils, independent of smoking. In this context, the findings of Tomlinson and colleagues are of interest [14]. They found a reduced short-term response to inhaled beclomethasone in current-smokers with asthma, which could be overcome by increasing the dose of beclomethasone from 400 μg/day to 2000 μg/day. It is tempting to speculate that the blunted corticosteroid treatment response in ex- and current-smokers can also be overcome by prolonged treatment, although this remains to be formally demonstrated in future prospective studies.

We did not find any differences in the level of lung function or severity of bronchial hyperresponsiveness between ex-, current- and never-smokers at baseline. However, we did observe a lower level of eosinophilic inflammation in blood and sputum and higher blood neutrophil counts in current-smokers than in never-smokers. These findings are consistent with earlier studies [7-12]. Additionally, we demonstrated that the level of eosinophilic inflammation was also lower in ex-smokers and very similar to that seen in current-smokers. To date, only one other study, also from our research group, reported on the inflammatory profile in ex-smoking asthmatics [23]. This study demonstrated that ex-smoking asthmatics have lower percentages of eosinophils in airway wall biopsies than never-smokers and that the percentage of sputum neutrophils is significantly higher in ex- than in never-smokers. The above findings suggest that smoking does not only have an acute effect on airway inflammation, but also a chronic effect that may persist for years after smoking cessation.

More severe neutrophilic inflammation in asthma has been associated with a reduced corticosteroid treatment response [24,25]. Therefore, the shift from eosinophilic to neutrophilic inflammation that we observed in ex- and current-smokers may be a possible explanation for the reduced short-term corticosteroid treatment response in ex- and current-smokers. Support for the hypothesis that the type of inflammation in ex- and current-smokers influences the corticosteroid treatment response is provided by our observation that smoking status was not independently associated with improvement in FEV_1_ %predicted, whereas less eosinophilic inflammation in sputum and blood was independently associated with lower improvement in FEV_1_ %predicted, both after 2-week and after 1-year ICS treatment. In addition, higher levels of blood neutrophils were also independently associated with lower improvement in FEV_1_ %predicted after 2-week ICS treatment. Interestingly, after 1-year ICS treatment there were no longer any significant differences in inflammation between ex-, current- or never-smokers (Additional file 1: Table S5). This suggests that long-term ICS treatment is able to correct the inflammatory differences in ex- and current-smokers, thereby normalizing their ICS treatment response. Other possible explanations for a lower corticosteroid responsiveness in ex- and current-smokers are epigenetic changes, e.g. reduced expression of histone deacetylases (HDAC) [26] and DNA methylation [27], more expression of the less active β isoform of the glucocorticoid receptor [28-30] and increased expression of pro-inflammatory transcription factors, such as nuclear factor-kappa B and activator protein 1 [31,32]. Finally, NO in cigarette smoke reduces the affinity of the glucocorticoid receptor for corticosteroids and reduces the binding of corticosteroids to the glucocorticoid receptor [33].

There are several strengths to our study. Our patients were extensively characterized, including lung function, bronchial hyperresponsiveness and inflammation in sputum and blood, at baseline and after 2-weeks and 1-year treatment with ICS. Our study also has some limitations. First, we performed post-hoc analyses on data from a study that was not originally designed to investigate the effects of smoking on inflammation or corticosteroid treatment response. Our study was originally a three-arm study (Figure 1). However, in the 2-week treatment analyses we included only patients treated with ICS, and in the 1-year treatment analyses we excluded one group of patients who were treated according to a program with step-down and eventually complete discontinuation of corticosteroids, which is not in agreement with the current guidelines. Due to this study design, the short- and long-term corticosteroid response was not investigated in the same groups. In this context, it is important to mention that the randomization of the study was performed with minimization for smoking status, age, previous dose of ICS, FEV_1_ %predicted, reversibility after 200 μg of salbutamol, PC_20_ methacholine, and serum IgE. This minimization ensures comparable treatment arms with minimal baseline differences. Second, current- and never-smokers were significantly younger than ex-smokers and therefore we had to adjust for age in all analyses.

## Conclusions

In conclusion, ex- and current-smokers have a different type of inflammation with less eosinophils and more neutrophils in their blood and sputum. These differences in the type of inflammation were present even several years after smoking cessation. Although we agree with the literature that ex- and current-smokers have a blunted short-term response to ICS, we did not find a difference in their long-term treatment response. Therefore, they should not be withheld from ICS treatment.

## Competing interests

E.D Telenga has no conflicts to declare. The University of Groningen received funding for research by Prof. H.A.M. Kerstjens from the following manufacturers of inhaled corticosteroids: GlaxoSmithKline, the manufacturer of beclometasone and fluticasone; AstraZeneca, the manufacturer of budesonide; and Nycomed, the manufacturer of ciclesonide. The University of Groningen received funding for research by Dr. N. H. T. ten Hacken from Boehringer Ingelheim, GSK, AstraZeneca, Nycomed and Chiesi. He has been consultant to Chiesi. The University of Groningen received funding for research by Prof. D. S. Postma from AstraZeneca, GSK, Nycomed. Travel to ERS or ATS has been partially funded by AstraZeneca, GSK, Chiesi, Nycomed. She has been consultant to AstraZeneca, Boehringer Ingelheim, Chiesi, GSK, Nycomed, TEVA. The University of Groningen received a research grant for research by dr. M. van den Berge from GlaxoSmithKline and Chiesi.

## Authors’ contributions

EDT Performed analysis, wrote the manuscript and approved the final version of the manuscript. HMK. Supervised the original study, critically revised the manuscript and approved the final version of the manuscript. NHTTH Critically revised the manuscript and approved the final version of the manuscript. DSP Supervised the original study, critically revised the manuscript and approved the final version of the manuscript. MB Supervised analysis, co-authored the manuscript and approved the final version of the manuscript.

## Pre-publication history

The pre-publication history for this paper can be accessed here:

http://www.biomedcentral.com/1471-2466/13/58/prepub

## Supplementary Material

Additional file 1: Table S1 Association between the amount of smoke exposure, as reflected by the number of cigarettes smoked daily and number of packyears and improvement in clinical and inflammatory variables after 2-week ICS treatment. **Table S2.** Association between the amount of smoke exposure, as reflected by the number of cigarettes smoked daily and number of packyears and clinical and inflammatory variables after 1-year ICS treatment. **Table S3.** Independent associations between improvement in FEV_1_ % predicted after 2-week or 1-year ICS treatment and smoking status and sputum eosinophil and neutrophil percentages. **Table S4.** Independent associations between improvement in FEV_1_ % predicted after 2-week or 1-year ICS treatment and smoking status and blood eosinophil and neutrophil levels. **Table S5.** Differences in clinical and inflammatory variables between ex-, current- and never-smokers at baseline. Click here for file
