# Supplementary material for: Inflammation and corticosteroid responsiveness in ex-, current- and never-smoking asthmatics
Source: BMC Pulm Med. 2013 Sep 22;13:58. doi: 10.1186/1471-2466-13-58 (PMC3849864; doi:10.1186/1471-2466-13-58)
Supplement: Additional file 1: Table S1 — Association between the amount of smoke exposure, as reflected by the number of cigarettes smoked daily and number of packyears and improvement in clinical and inflammatory variables after 2-week ICS treatment. Table S2. Association between the amount of smoke exposure, as reflected by the number of cigarettes smoked daily and number of packyears and clinical and inflammatory variables after 1-year ICS treatment. Table S3. Independent associations between improvement in FEV1 % predicted after 2-week or 1-year ICS treatment and smoking status and sputum eosinophil and neutrophil percentages. Table S4. Independent associations between improvement in FEV1 % predicted after 2-week or 1-year ICS treatment and smoking status and blood eosinophil and neutrophil levels. Table S5. Differences in clinical and inflammatory variables between ex-, current- and never-smokers at baseline. [file 1471-2466-13-58-S1.doc]

**Additional file 1**

Self-management plan

PB= personal best

1. If PEF ≥80% of PB, continue maintenance treatment: bronchodilators as needed and same dose of fluticasone twice daily
2. If PEF <80% and >60% of PB, double the dose of inhaled fluticasone for one month. If after one month PEF is ≥80% of PB and symptoms are similar to baseline then return to previous dose. If PEF is still below 80% of PB then again double the dose of inhaled fluticasone for one month (therefore 4 times the original dose). If PEF is ≥80% of PB but symptoms are not back to baseline then continue with this dose.
3. If PEF <60% of PB, double the dose of inhaled fluticasone for one month and start with a course of oral prednisolone 30 mg/day for 7 days. After one month, if PEF is ≥80% and symptoms are similar to baseline then return to previous dose.
4. If PEF is still below 80% of PB then again double the dose of inhaled fluticasone for one month (therefore 4 times the original dose). If PEF is ≥80% of PB but symptoms are not back to baseline then continue with this dose.
5. If symptoms increase with 1 on a scale of 4 during more than 1 day, even in case PEF ≥80% of PB, double the dose of inhaled fluticasone and start with a course of oral prednisolone 30 mg/day for 7 days. After one month if symptoms are similar to baseline then return to previous dose. If symptoms are not back to baseline, again double the dose of inhaled fluticasone and start with a course of oral prednisolone (30 mg/day) for 7 days.

Symptom score

*How was your asthma during the day?*

0 = no asthma, normal unrestricted activity

1 = Wheezing or short of breath on strenuous exercise/hurrying, otherwise asthma not unduly troublesome.

2 = Wheezing or short of breath most of the day- normal activities difficult

3 = Asthma bad- could not go to work or to do household or carrying out usual activities at all because of short of breath

How was your asthma at night?

0 = Good night, slept well, no asthma

1 = Good night, slept well, but woke once with wheeze and cough

2 = Woken 2-3 times by cough/ wheeze/breathlessness/asthma

3 = Bad night, awake most of the night with cough/wheeze/breathlessness/asthma

**Additional Table S1** Association between the amount of smoke exposure, as reflected by the number of cigarettes smoked daily and number of packyears and improvement in clinical and inflammatory variables after 2-week ICS treatment

|  | Cigarettes/day | | Packyears | |
| --- | --- | --- | --- | --- |
|  | b | p-value | b | p-value |
| ΔFEV_1_ (L) | -0.01 | 0.528 | -0.01 | 0.529 |
| ΔFEV_1_ (%predicted) | -0.48 | 0.242 | -0.55 | 0.025 |
| ΔReversibility (% of the predicted FEV_1_) | -0.26 | 0.199 | -0.13 | 0.394 |
| ΔPC_20_ methacholine  (doubling concentrations) | -0.06 | 0.365 | 0.00 | 0.987 |
| ΔPC_20_ AMP (doubling concentrations) | -0.14 | 0.342 | 0.08 | 0.502 |
| ΔSputum eosinophils (%) * | 0.04 | 0.288 | 0.01 | 0.812 |
| ΔBlood eosinophils (10^9^/L) * | 0.00 | 0.917 | 0.00 | 0.804 |
| ΔSputum ECP (μg/L) * | 0.05 | 0.282 | 0.03 | 0.387 |
| ΔSerum ECP (μg/L) * | 0.02 | 0.328 | 0.01 | 0.763 |
| ΔSputum neutrophils (%) * | 0.00 | 0.924 | 0.00 | 0.850 |
| ΔBlood neutrophils (10^9^/L) * | -0.00 | 0.915 | 0.00 | 0.950 |
| ΔExhaled NO (ppb) | 0.08 | 0.788 | 0.18 | 0.367 |

b = unstandardized regression coefficient, ICS = inhaled corticosteroids, FEV_1_ = forced expiratory volume in one second, PC_20_ = provocative concentration causing a 20% fall in FEV_1_, AMP = adenosine-5’-monophosphate, ECP = eosinophilic cationic protein, NO = nitric oxide, * variable log-transformed.

**Additional Table S2** Association between the amount of smoke exposure, as reflected by the number of cigarettes smoked daily and number of packyears and clinical and inflammatory variables after 1-year ICS treatment

|  | Cigarettes/day | | Packyears | |
| --- | --- | --- | --- | --- |
|  | b | p-value | b | p-value |
| ΔFEV_1_ (L) | -0.02 | 0.349 | -0.03 | 0.034 |
| ΔFEV_1_ %pred | -0.42 | 0.437 | -0.66 | 0.032 |
| ΔPC_20_ methacholine (doubling concentrations) | -0.02 | 0.730 | -0.14 | 0.043 |
| ΔSputum eosinophils (%) * | 0.07 | 0.600 | 0.04 | 0.752 |
| ΔBlood eosinophils (10^9^/L) * | -0.02 | 0.446 | -0.02 | 0.281 |
| ΔSputum ECP (μg/L) * | 0.02 | 0.577 | 0.02 | 0.718 |
| ΔSerum ECP (μg/L) * | 0.03 | 0.305 | 0.05 | 0.015 |
| ΔSputum neutrophils (%) * | 0.03 | 0.179 | -0.01 | 0.686 |
| ΔBlood neutrophils (10^9^/L) * | -0.02 | 0.599 | -0.01 | 0.832 |
| ΔExhaled NO (ppb) | 0.03 | 0.917 | -0.08 | 0.670 |

b = unstandardized regression coefficient, ICS= inhaled corticosteroids, FEV_1_ = forced expiratory volume in one second, PC_20_ = provocative concentration causing a 20% fall in FEV_1_, ECP = eosinophilic cationic protein, NO = nitric oxide, ppb = parts per billion, * variable log-transformed.

**Additional Table S3** Independent associations between improvement in FEV_1_ % predicted after 2-week or 1-year ICS treatment and smoking status and sputum eosinophil and neutrophil percentages

|  | 2 week | | 1 year | |
| --- | --- | --- | --- | --- |
|  | b | p-value | b | p-value |
| Sputum eosinophils (%) * | 0.252 | 0.005 | 0.232 | 0.002 |
| Sputum neutrophils (%) * | -0.133 | 0.173 | -0.021 | 0.828 |
| Ex- vs. never-smokers | -0.203 | 0.170 | -0.084 | 0.542 |
| Current- vs. never-smokers | -0.184 | 0.229 | 0.002 | 0.990 |
| Baseline FEV_1_ (%) | -0.212 | 0.003 | -0.179 | 0.044 |

b = unstandardized regression coefficient, ICS= inhaled corticosteroids, FEV_1_ = forced expiratory volume in one second, * variable log-transformed.

**Additional Table S4** Independent associations between improvement in FEV_1_ % predicted after 2-week or 1-year ICS treatment and smoking status and blood eosinophil and neutrophil levels

|  | 2 week | | 1 year | |
| --- | --- | --- | --- | --- |
|  | b | p-value | b | p-value |
| Blood eosinophils (10^9^/L) * | 0.529 | 0.022 | 0.469 | 0.025 |
| Blood neutrophils (10^9^/L) * | -0.343 | 0.049 | -0.207 | 0.288 |
| Ex- vs. never-smokers | -0.256 | 0.086 | -0.062 | 0.680 |
| Current- vs. never-smokers | -0.226 | 0.148 | 0.036 | 0.825 |
| Baseline FEV_1_ (%) | -0.280 | 0.000 | -0.193 | 0.044 |

b = unstandardized regression coefficient, ICS= inhaled corticosteroids, FEV_1_ = forced expiratory volume in one second, * variable log-transformed.

**Additional Table S5** Differences in clinical and inflammatory variables between ex-, current- and never-smokers at baseline

|  | **Ex-smokers**  (n=21) | **Current-smokers**  (n=17) | **Never-smokers**  (n=37) | **p-value** |
| --- | --- | --- | --- | --- |
| Treatment ^#^  (self management / FP500) | 10 / 11 | 11 / 6 | 17 / 20 |  |
| FEV_1_ (L) | 3.4 (2.6, 3.6) | 3.0 (2.5, 3.7) | 3.3 (3.2, 3.7) | 0.15 |
| FEV_1_ (%predicted) | 85 (80, 90) | 80 (72, 97) | 91 (84, 102) | 0.55 |
| PC_20_ methacholine (mg/ml) ^§^ | 8.8 (0.05, 39) | 3.6 (0.14, 39) | 6.5 (0.04, 39) | 0.46 |
| Sputum eosinophils (%) | 0.7 (0.0, 1.8) | 0.3 (0.0, 5.4) | 1.3 (0.0, 3.5) | 0.75 |
| Blood eosinophils (10^3^/µL) | 0.25 (0.13, 0.37) | 0.24 (0.15, 0.40) | 0.31 (0.19, 0.45) | 0.26 |
| Sputum ECP (μg/L) | 49 (18, 107) | 34 (27, 103) | 26 (14, 47) | 0.50 |
| Serum ECP (μg/L) | 10 (8, 17) | 13 (7, 21) | 13 (9, 17) | 0.50 |
| Sputum neutrophils (%) | 38 (23, 53) | 46 (34, 58) | 32 (23, 62) | 0.94 |
| Blood neutrophils (10^3^/µL) | 3.5 (3.1, 4.7) | 4.1 (3.5, 5.0) | 3.2 (5.6, 3.7) | 0.44 |
| Exhaled NO (ppb) | 10.2 (4.5, 16.5) | 8.0 (5.2, 10.5) | 8.9 (6.7, 15.9) | 0.21 |

Values are presented as medians with interquartile ranges, unless stated otherwise, # = number, § geometric mean (range), self management = treatment according to self management plan in the online supplemental appendix, FP500 = fluticasone propionate 500 µg/day, FEV_1_ = forced expiratory volume in one second, PC_20_ = provocative concentration causing a 20% fall in FEV_1_.
